# Vidarabine, an anti-herpes agent, improves *Porphyromonas gingivalis* lipopolysaccharide-induced cardiac dysfunction in mice

**DOI:** 10.1186/s12576-023-00873-5

**Published:** 2023-08-09

**Authors:** Michinori Tsunoda, Ichiro Matsuo, Yoshiki Ohnuki, Kenji Suita, Misao Ishikawa, Takao Mitsubayashi, Aiko Ito, Yasumasa Mototani, Kenichi Kiyomoto, Akinaka Morii, Megumi Nariyama, Yoshio Hayakawa, Kazuhiro Gomi, Satoshi Okumura

**Affiliations:** 1 Department of Physiology, Tsurumi University School of Dental Medicine, 2-1-3 Tsurumi, Tsurumi-ku, Yokohama, 230-8501 Japan; 2 Department of Periodontology, Tsurumi University School of Dental Medicine, Yokohama, 230-8501 Japan; 3 Department of Oral Anatomy, Tsurumi University School of Dental Medicine, Yokohama, 230-8501 Japan; 4 Department of Orthodontology, Tsurumi University School of Dental Medicine, Yokohama, 230-8501 Japan; 5 Department of Pediatric Dentistry, Tsurumi University School of Dental Medicine, Yokohama, 236-8501 Japan; 6 Department of Dental Anesthesiology, Tsurumi University School of Dental Medicine, Yokohama, 230-8501 Japan

**Keywords:** β-Adrenergic signaling, Periodontitis, Adenylyl cyclase, Apoptosis, Fibrosis, Signal transduction, Heart failure

## Abstract

In this work, we examined the involvement of type 5 adenylyl cyclase (AC5) in cardiac dysfunction induced in mice given *Porphyromonas gingivalis* lipopolysaccharide (PG-LPS) at a dose equivalent to the circulating levels in periodontitis (PD) patients. Cardiac function was significantly decreased in mice given PG-LPS compared to the control, but treatment for 1 week with the AC5 inhibitor vidarabine ameliorated the dysfunction. Cardiac fibrosis and myocyte apoptosis were significantly increased in the PG-LPS group, but vidarabine blocked these changes. The PG-LPS-induced cardiac dysfunction was associated with activation of cyclic AMP/Ca^2+^-calmodulin-dependent protein kinase II signaling and increased phospholamban phosphorylation at threonine 17. These results suggest that pharmacological AC5 inhibition may be a promising approach to treat PD-associated cardiovascular disease.

**Supplementary Information:**

The online version contains supplementary material available at 10.1186/s12576-023-00873-5.

## Background

Oral frailty is defined by the Japan Dental Association as a decrease in oral function accompanied by a decrease in mental and physical functions [1]. A recent cohort study with 2044 elderly Japanese subjects found that people with oral frailty were at higher risk of physical frailty requiring nursing care, as well as death, than were those without oral frailty [2]. Interestingly, a large-scale epidemiological survey found a higher risk of oral health problems among cardiovascular disease (CVD) patients than among community-dwelling populations [3, 4]. Poor oral health, as exemplified by periodontitis (PD), missing teeth and loss of dental occlusal capability, was associated with a more than two-fold increase in the risk of future CVD [3, 4]. Moreover, the prevalence of malnutrition among patients with CVD is high because of anorexia or intestinal edema, cytokine-induced catabolism, and cardiac cachexia [5]. However, the longitudinal association between oral health problems and CVD is not yet clearly understood.

CVD is a major cause of physical frailty and mortality, and chronic stimulation of the sympathetic nervous system is a common cause of CVD in patients [6]. Adenylyl cyclase (AC) is the target enzyme of β-adrenergic receptor (β-AR) signaling stimulation [7]. There are nine major mammalian isoforms of AC, with type 5 AC (AC5) being the major cardiac isoform in adults [8–10]. We have developed a mouse model with disruption of AC5 [11–13] and we have also identified the antiviral agent vidarabine as an inhibitor of cardiac AC in mice [14]. Building on that work, we found that genetic and pharmacological AC5 inhibition might be associated with resistance to the development of CVD and increased longevity [15]. We also recently presented evidence that occlusal disharmony-induced cardiac fibrosis and cardiac myocyte apoptosis might be caused by reactive oxygen species (ROS) production derived from nicotinamide adenine dinucleotide phosphate oxidase 4 (NOX4) via activation of AC5 in mice. The activity of NOX4 is regulated by its expression level [16], so in this work, we examined NOX4 protein expression in the heart of mice given *Porphyromonas gingivalis* lipopolysaccharide (PG-LPS) with or without vidarabine.

PD is a serious oral health problem, which can even lead to tooth loss and loss of dental occlusal capability, and its treatment is associated with improved resistance to the development of CVD and increased longevity due to reduction of oxidative stress [17, 18]. In this context, we have demonstrated that persistent subclinical exposure to PG-LPS induces myocardial cell damage and heart failure with the activation of cyclic AMP (cAMP)-protein kinase A (PKA) and Ca^2+^/calmodulin-dependent protein kinase II (CaMKII) signaling [18]. Since phosphorylation of most Ca^2+^-handling proteins is altered in many models of experimental heart failure and this might lead to increased Ca^2+^ leakage, we also examined the effects of PG-LPS treatment on the phosphorylation of phospholamban (PLN) at Thr-17, which is known to be mediated by CaMKII [19].

The role of AC5 in PG-LPS-mediated cardiac remodeling and dysfunction remains poorly understood. However, previous reports on AC5 deficiency in mice and PG-LPS-treated mice suggest that inhibition of cardiac AC subtypes with vidarabine might prevent myocardial cell damage and heart failure in mice treated with PG-LPS at a dose equivalent to the level seen in subclinical PD. Importantly, vidarabine has been used as an anti-viral drug for many years in humans [14]. Therefore, vidarabine, rather than a β-blocker, might be a safe and immediately clinically available drug for the treatment or prevention of cardiac dysfunction induced by PG-LPS.

Thus, the aim of this study was to examine the effects of AC5 inhibition with vidarabine on cardiac function, cardiac fibrosis and myocyte apoptosis in mice treated with PG-LPS at a dose equivalent to the circulating levels in PD patients, and to clarify the mechanisms involved.

## Materials and methods

### Mice and experimental protocol

All experiments were performed on male 12-week-old C57BL/6 mice obtained from CLEA Japan (Tokyo, Japan). Mice were group-hosed at 23 °C under a 12–12 light/dark cycle with lights on at 8:00 AM in accordance with standard conditions for mouse studies by our group [20–22]. Both food and water were available ad libitum.

**Fig. 1:**
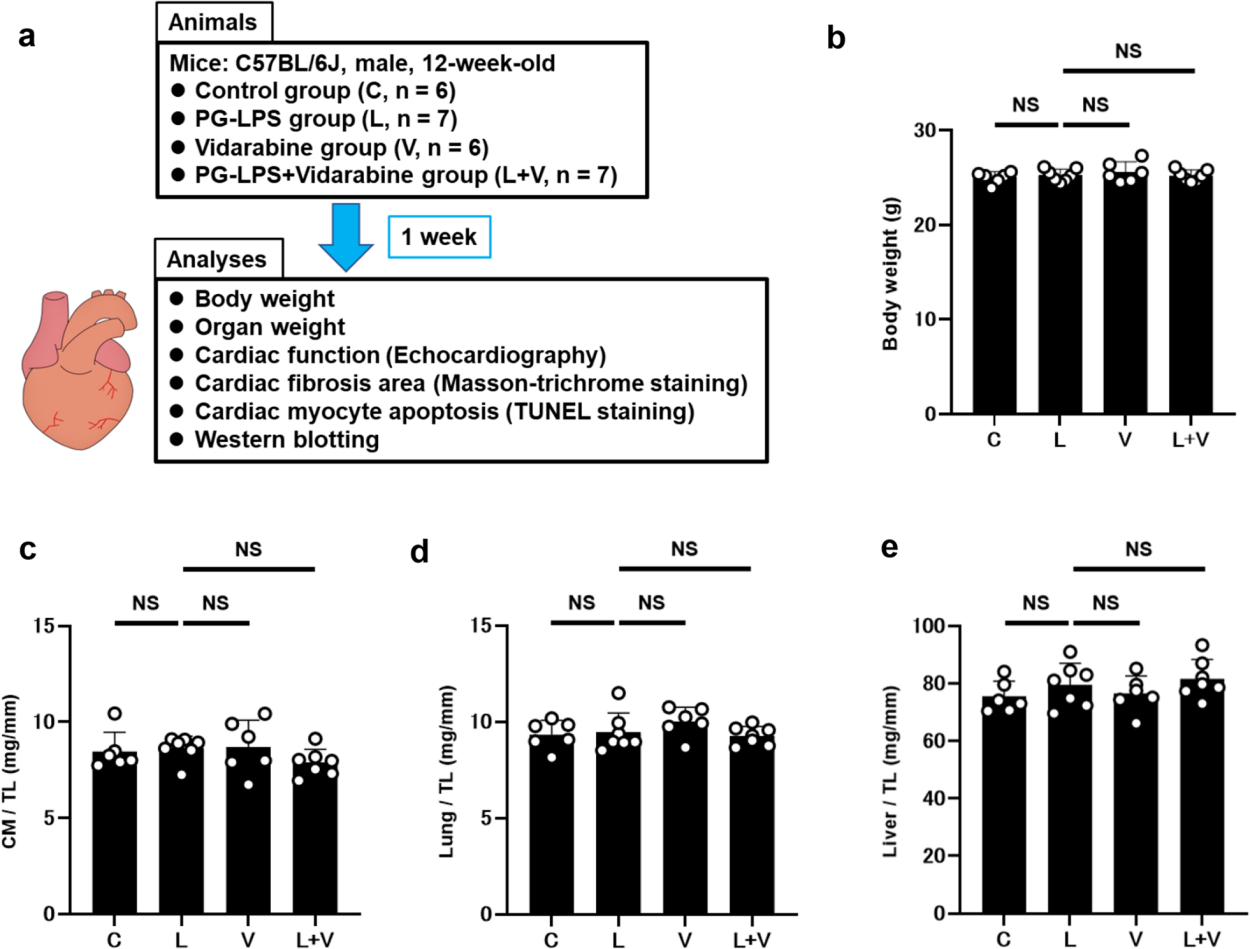
Schematic illustration of experimental procedure and comparison of body weight, cardiac muscle weight, lung weight and liver weight. **a** Male 12-week-old C57BL/6 mice were divided into four groups: a normal control group (Control), a PG-LPS-treated group (L), a vidarabine-treated group (V), and a PG-LPS plus vidarabine-treated (L + V) group. Chronic infusion of vidarabine was performed for 1-week at a dose of 15 mg/kg/day with the osmotic mini-pumps and the indicated measurements were made. **b** The Control, PG-LPS, vidarabine and PG-LPS plus vidarabine groups showed similar body weight. NS, not significantly different from the Control (*P* > 0.05). One-way ANOVA followed by Tukey’s post hoc test). **c** No significant difference in heart (**c**), lung (**d**) or liver size in terms of weight per tibial length (TL) ratio (mg/mm) at 1 week after PG-LPS infusion among the four groups. *P* = NS, not significantly different from the Control. One-way ANOVA followed by the Tukey’s post hoc test). Data are presented as mean ± SD and scattered dots show individual data

PG-LPS (#14966-71; Invivogen, San Diego, CA, USA) was dissolved in phosphate-buffered saline (PBS, pH = 7.5) to prepare a 0.6 mg/mL stock solution [23], and appropriate volumes of this solution according to the desired dose (PG-LPS: 0.8 mg/kg) were added to 0.2 mL of PBS to prepare the solution for intraperitoneal (i.p.) injection (once daily for 1 week). Mice were group-housed (approximately 3 per cage) and were divided into four groups: a normal control group (Control), a PG-LPS treatment group, a vidarabine-only treatment group, and a PG-LPS plus vidarabine treatment group (PG-LPS + vidarabine) (Fig. 1a). Chronic infusion of vidarabine dissolved in DMSO (#359-13471; Sigma, St. Lois MO, USA) was performed for 1 week at a dose of 15 mg/kg/day, delivered with osmotic mini-pumps (Model 2002; ALZET, Cupertino, CA, USA) [15, 21, 24]. The dose of vidarabine (15 mg/kg/day; a dose approved for clinical use in humans) was selected based upon that used in previous studies: this dose did not eliminate the inotropic effects of acute isoproterenol, did not depress cardiac function at baseline, and retained high selectivity for AC5 [15]. Body weight (BW) was monitored throughout the 1-week experimental period (Control: *n* = 6, PG-LPS: *n* = 7, vidarabine: *n* = 6, PG-LPS + vidarabine: *n* = 7) (Fig. 1b). The dose of PG-LPS used in this study is consistent with the circulating levels in PD patients, indicating that this model is not a sepsis model, and indeed, no mortality was observed [23]. After the completion of treatment, mice were anesthetized with isoflurane (1.0–1.5% v/v) and killed by cervical dislocation [25]. The heart, lungs and liver were excised, weighed, frozen in liquid nitrogen, and stored at − 80 °C. The ratios of organ mass (mg) to tibial length (TL; mm) were used as indexes of organ volume (Fig. 1c–e). All animal experiments complied with the ARRIVE guidelines [26] and were carried out in accordance with the National Institutes of Health guide for the care and use of laboratory animals [27] and institutional guidelines.

### Physiological experiments

Mice were anesthetized with isoflurane vapor (1.0–1.5% v/v) titrated to maintain the lightest anesthesia possible and echocardiographic measurements were performed by means of ultrasonography (TUS-A300, Toshiba, Tokyo, Japan) as described previously [19].

All left ventricular (LV) dimensions are presented as the average of four consecutive selected beats. Heart rate (HR) was determined from the cardiac cycles recorded on the M-mode tracing, using at least three consecutive beats. The other parameters were calculated from M-mode-derived LV dimensions using the Teichholz formula [28]:

$$ {\text{EDV }} = \, \left( {{7 } \times {\text{ LVIDd}}^{{3}} /{1}000} \right)/\left( {{2}.{4 } + \, \left( {{\text{LVIDd}}/{1}0} \right)} \right){\text{ and ESV}} = \left( {{7 } \times {\text{ LVIDs}}^{{3}} /{1}000} \right)/\left( {{2}.{4} + \left( {{\text{LVIDd}}/{1}0} \right)} \right)\left( {{\text{mL}}} \right) $$

EDV (mL): left ventricular end-diastolic volume; ESV (mL): left ventricular end-systolic volume; LVIDd (mm): left ventricular internal dimension at end-diastole; LVIDs (mm): left ventricular internal dimension at end-systole.

$$ {\text{Stroke volume }}\left( {{\text{SV}}} \right) = {\text{EDV}} - {\text{ESV}}\left( {{\text{mL}}} \right) $$

$$ {\text{Cardiac output }}\left( {{\text{CO}}} \right) = {\text{HR}} \times {\text{SV }}\left( {{\text{ml}}/{\text{min}}} \right) $$

$$ {\text{Left ventricular ejection fraction}}\,\left( {{\text{EF}}} \right) = {1}00 \times {\text{SV}}/{\text{EDV }}\left( \% \right) $$

$$ {\text{Left ventricular fractional shortening}}\,\left( {\% {\text{FS}}} \right) = 100 \times \left( {{\text{LVIDd }} - {\text{ LVIDs}}} \right)/{\text{LVIDd }}\left( \% \right) $$

All LV dimensions calculated using the Teichholz formula in wild-type control (12-week-old C57BL/6 mice) were consistent with those reported in previous studies by us [20] and another group [29].

### Evaluation of fibrosis

Cross Sects. (10 μm) (Control; *n* = 6; PG-LPS; *n* = 7; vidarabine; *n* = 6; PG-LPS + vidarabine; *n* = 6) were cut with a cryostat (CM1900, Leica Microsysytems, Nussloch, Germany) at − 20 °C. The sections were air-dried and fixed with 4% paraformaldehyde (v/v) in 0.1 M PBS [30–32].

Interstitial fibrosis was evaluated by Masson-trichrome staining using an Accustatin Trichrome Stain Kit (#HT15-1KT; Sigma) in accordance with the manufacturer’s protocol, as described previously [19, 33]. Interstitial fibrotic regions were quantified using image analysis software (Image J 1.45) to evaluate the percentage of blue area in the Masson-trichrome section [19].

### Evaluation of apoptosis

Apoptosis was determined by terminal deoxyribonucleotidyl transferase (TdT)-mediated biotin-16-deoxyuridine triphosphate (dUTP) nick-end labeling (TUNEL) staining using the Apoptosis in situ Detection Kit (#293-71501; Wako, Osaka, Japan). TUNEL-positive nuclei per field of view were manually counted in six sections of four groups (Control; *n* = 4; PG-LPS; *n* = 4; vidarabine; *n* = 4; PG-LPS + vidarabine; *n* = 5) over a microscopic field of 20×, averaged and expressed as the ratio of TUNEL-positive nuclei (%) [12, 19]. Limiting the counting of total nuclei and TUNEL-positive nuclei to areas with a true cross section of myocytes made it possible to selectively count only those nuclei that were clearly located within myocytes.

### Western blotting

The cardiac muscle excised from the mice (Control; *n* = 6; PG-LPS; *n* = 7; vidarabine; *n* = 6; PG-LPS + vidarabine; *n* = 7) (Fig. 1a) was homogenized in a Polytron (Kinematica AG, Lucerne, Switzerland) in ice-cold RIPA buffer (#89900; Thermo Fisher Scientific, Waltham, MA, USA: 25 mM Tris–HCl (pH 7.6), 150 mM NaCl, 1% NP-40, 1% sodium deoxycholate, 0.1% SDS) with addition of Halt™ Protease Inhibitor Cocktail, EDTA-free (#87785; Thermo Fisher Scientific), and the homogenate was centrifuged at 13,000*g* for 10 min at 4 °C. The supernatant was collected and the protein concentration was measured using a DC protein assay kit (Bio-Rad, Hercules, CA, USA). Equal amounts of protein (5 μg) were subjected to SDS–polyacrylamide gel electrophoresis and blotted onto 0.2 mm PVDF membrane (Millipore, Billerica, MA, USA).

**Fig. 2:**
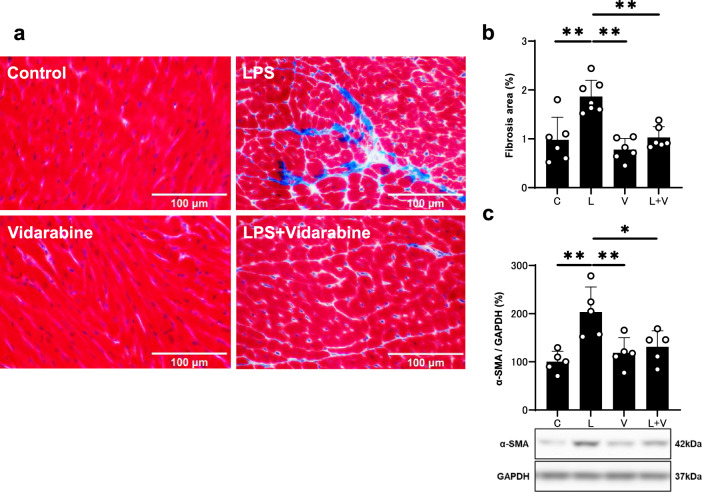
Effects of vidarabine on PG-LPS-induced fibrosis in the heart. **a** Representative images of Masson-trichrome-stained sections of cardiac muscle in the Control (*upper left*), PG-LPS (*upper right*), vidarabine (*lower left*), and BO + vidarabine (*lower right*) groups. Scale bar: 100 μm. **b** The area of fibrosis was significantly increased in the PG-LPS group (*n* = 7, **P < 0.01), but this increase was blocked in the PG-LPS + vidarabine group (*n* = 6, **P < 0.01). One-way ANOVA followed by Tukey’s post hoc test). **c** Expression of α-SMA, a fibrosis-related gene, was significantly increased in the cardiac muscle of PG-LPS group (*n* = 5, **P < 0.01), but this increase was blocked in the cardiac muscle of PG-LPS + vidarabine group (*n* = 5, *P < 0.05). One-way ANOVA followed by Tukey’s post hoc test). Full-size images of immunoblots are presented in Additional file 1: Fig. S1. Data are presented as mean ± SD and scattered dots show individual data

Western blotting was conducted with commercially available antibodies [11, 12, 19] directed against α-smooth muscle actin (α-SMA) (1:1000, #19245), CaMKII (1:1000, #3362), phospho-CaMKII (1:1000, Thr-286, #3361) and B cell lymphoma 2 (BCL-2) (1:1000, #3498) [from Cell Signaling Technology (Boston, MA, USA)], glyceraldehyde 3-phosphate dehydrogenase (GAPDH) (1:200, sc-32233) [from Santa Cruz Biotechnology (Santa Cruz, CA, USA)], phospho-phospholamban (PLN) (1:5000, Thr-17, #A010-13) and PLN (1:5000, #A010-14) [from Badrilla (Leeds, UK)], NOX4 (1:1000, #ab133303) [from Abcam (Cambridge, UK)], AC5 (1:1000, #SAB4500206) [from Sigma] and oxidized CaMKII (Met-281/282) (1:1000, #07-1387) [from Millipore (Billerica, MA, USA)]. Horseradish peroxide-conjugated anti-rabbit (1:5000, #NA934) or anti-mouse IgG (1:5000, #NA931) purchased from GB Healthcare (Amersham, UK) was used as a secondary antibody. The primary and secondary antibodies were diluted in Tris-buffered saline (pH 7.6) with 0.1% Tween 20 and 5% bovine serum albumin. The blots were visualized with enhanced chemiluminescence solution (ECL: Prime Western Blotting Detection Reagent) and scanned with a densitometer (ASL-600, GE Healthcare, Piscataway, NJ, USA, or LAS-1000, Fuji Photo Film, Tokyo, Japan). Note that there are different numbers of samples in different western blotting figures (Figs. 2c, 4a–e) because we excluded outliers (extremely low or high values, compared to others in the same group).

### Statistical analysis

Data show means ± standard deviation (SD). Comparison of data was performed using one-way ANOVA followed by Tukey’s post hoc test. Differences were considered significant when *P* < 0.05.

## Results

### Effect of PG-LPS on BW and size of heart, lung and liver with/without vidarabine

The Control, PG-LPS, vidarabine, PG-LPS + vidarabine groups all showed similar BW at 1 week after the PG-LPS infusion (PG-LPS [*n* = 7]: 25.2 ± 0.7, vidarabine [*n* = 6]: 25.6 ± 1.1, PG-LPS + vidarabine [*n* = 7]: 25.2 ± 0.6 g, all not significantly different [NS; *P* > 0.05] vs. Control [*n* = 6; 25 ± 0.6 g]) (Fig. 1b).

We also examined the effects of PG-LPS with/without vidarabine on heart size in terms of cardiac muscle mass per TL ratio (mg/mm) (Fig. 1c) and the effects on wet lung and liver weight per TL (Fig. 1d, e). Similar results were obtained among the four groups.

Thus, neither PG-LPS nor vidarabine at the dose used in this experiment appeared to influence growth, cardiac hypertrophy or lung/liver congestion during the 1-week experimental period.

### Effects of PG-LPS on cardiac function with/without vidarabine

We conducted echocardiography (Table 1) to evaluate cardiac function in terms of EF and %FS. Both parameters were significantly decreased in the PG-LPS group compared to the control (EF: Control [*n* = 6] vs. PG-LPS [*n* = 7]: 67 ± 1.0 vs. 61 ± 0.9%, *P* < 0.01; %FS: Control [*n* = 6] vs. PG-LPS [*n* = 6]: 32 ± 0.8 vs. 28 ± 0.6%, *P* < 0.01). Vidarabine alone [*n* = 6] had no effect on EF and %FS, but blocked the PG-LPS-induced decrease of EF and %FS at 1 week (EF: PG-LPS [*n* = 7] vs. PG-LPS + vidarabine [*n* = 6]: 61 ± 0.9 vs. 67 ± 1.4%, *P* < 0.05; %FS: PG-LPS [*n* = 7] vs. PG-LPS + vidarabine [*n* = 6]: 28 ± 0.6 vs. 32 ± 0.9%, *P* < 0.05).

**Table 1:** Cardiac function assessed by echocardiography at 1 week after PG-LPS

	Control	LPS	Vid	LPS + Vid
n	6	7	6	6
EF	67 ± 1.0	61 ± 0.9**	66 ± 4.7^##^	67 ± 1.4^#^
EDV	0.23 ± 0.01	0.21 ± 0.01	0.22 ± 0.03	0.21 ± 0.01
ESV	0.08 ± 0.004	0.08 ± 0.005	0.08 ± 0.02	0.07 ± 0.006
%FS	32 ± 0.8	28 ± 0.6**	32 ± 3.1^##^	32 ± 0.9^#^
LVIDd	4.51 ± 0.08	4.37 ± 0.08	4.47 ± 0.22	4.41 ± 0.08
LVIDs	3.1 ± 0.06	3.1 ± 0.07	3.1 ± 0.24	3.0 ± 0.08
HR	420 ± 35	443 ± 27	441 ± 40	406 ± 42
SV	0.15 ± 0.008	0.13 ± 0.005*	0.15 ± 0.02	0.14 ± 0.007
CO	60 ± 7.0	52 ± 3.1	61 ± 9.8	57 ± 4.5
IVSTd	0.5 ± 0.03	0.5 ± 0.04	0.5 ± 0.04	0.5 ± 0.04
LVSTs	0.96 ± 0.07	0.91 ± 0.07	0.91 ± 0.03	0.94 ± 0.05
LVPWTd	0.52 ± 0.03	0.52 ± 0.02	0.53 ± 0.05	0.52 ± 0.03
LVPWTs	0.96 ± 0.04	0.88 ± 0.03*	0.93 ± 0.08	0.89 ± 0.04

EF (%): left ventricular ejection fraction; EDV (mL): left ventricular end-diastolic volume; ESV (mL): left ventricular end-systolic volume; %FS: % fractional shortening; LVIDd (mm): left ventricular internal dimension at end-diastole; LVIDs (mm): left ventricular internal dimension at end-diastole; HR (bpm): heart rate; SV (mL): stroke volume; CO (mL/min): cardiac output; IVSTd (mm): interventricular septum thickness at end-diastole. LVSTs (mm): interventricular septum thickness at end-systole; LVPWTd (mm): left ventricular posterior wall thickness at end-diastole. LVPWTs (mm): left ventricular posterior wall thickness at end-diastole

**P < 0.01 vs. Control, *P < 0.05 vs. Control ^##^*P* < 0.01 vs. LPS; ^#^*P* < 0.05 vs. LPS

These data suggest that PG-LPS treatment decreases cardiac function at least in part through the activation of AC5.

### Effects of PG-LPS on cardiac fibrosis with/without vidarabine

We examined the effects of PG-LPS with/without vidarabine on fibrosis in cardiac muscle by means of Masson-trichrome staining (Fig. 2a). PG-LPS treatment significantly increased the area of fibrosis in cardiac muscle (Control [*n* = 6] vs. PG-LPS [*n* = 7]: 0.98 ± 0.46 vs. 1.86 ± 0.34%, *P* < 0.01 by one-way ANOVA followed by Tukey’s post hoc test), in accordance with our previous findings [18, 20] (Fig. 2b). Vidarabine alone did not alter the area of fibrosis, but it blocked the PG-LPS-induced increase of fibrosis (PG-LPS [*n* = 6] vs. PG-LPS + vidarabine [*n* = 7]: 1.86 ± 0.34 vs. 1.03 ± 0.22%, *P* < 0.05 by one-way ANOVA followed by Tukey’s post hoc test) (Fig. 2b).

### Effects of PG-LPS on α-SMA expression with/without vidarabine

We also evaluated cardiac fibrosis by measuring the level of α-SMA expression at 1 week after the start of PG-LPS, because this parameter is closely associated with cardiac fibrosis [31, 34]. The expression level of α-SMA was significantly increased in cardiac muscle of PG-LPS-treated mice (Control [*n* = 5] vs. PG-LPS [*n* = 5]: 100 ± 22 vs. 203 ± 52%, *P* < 0.01 by one-way ANOVA followed by Tukey’s post hoc test), and the increase was significantly suppressed by vidarabine (PG-LPS [*n* = 5] vs. PG-LPS + vidarabine [*n* = 5]: 203 ± 52 vs. 131 ± 33%, *P* < 0.01 by one-way ANOVA followed by Tukey’s post hoc test) (Fig. 2c and Additional file 1: Fig. S1).

### Effects of PG-LPS on cardiac apoptosis with/without vidarabine

**Fig. 3:**
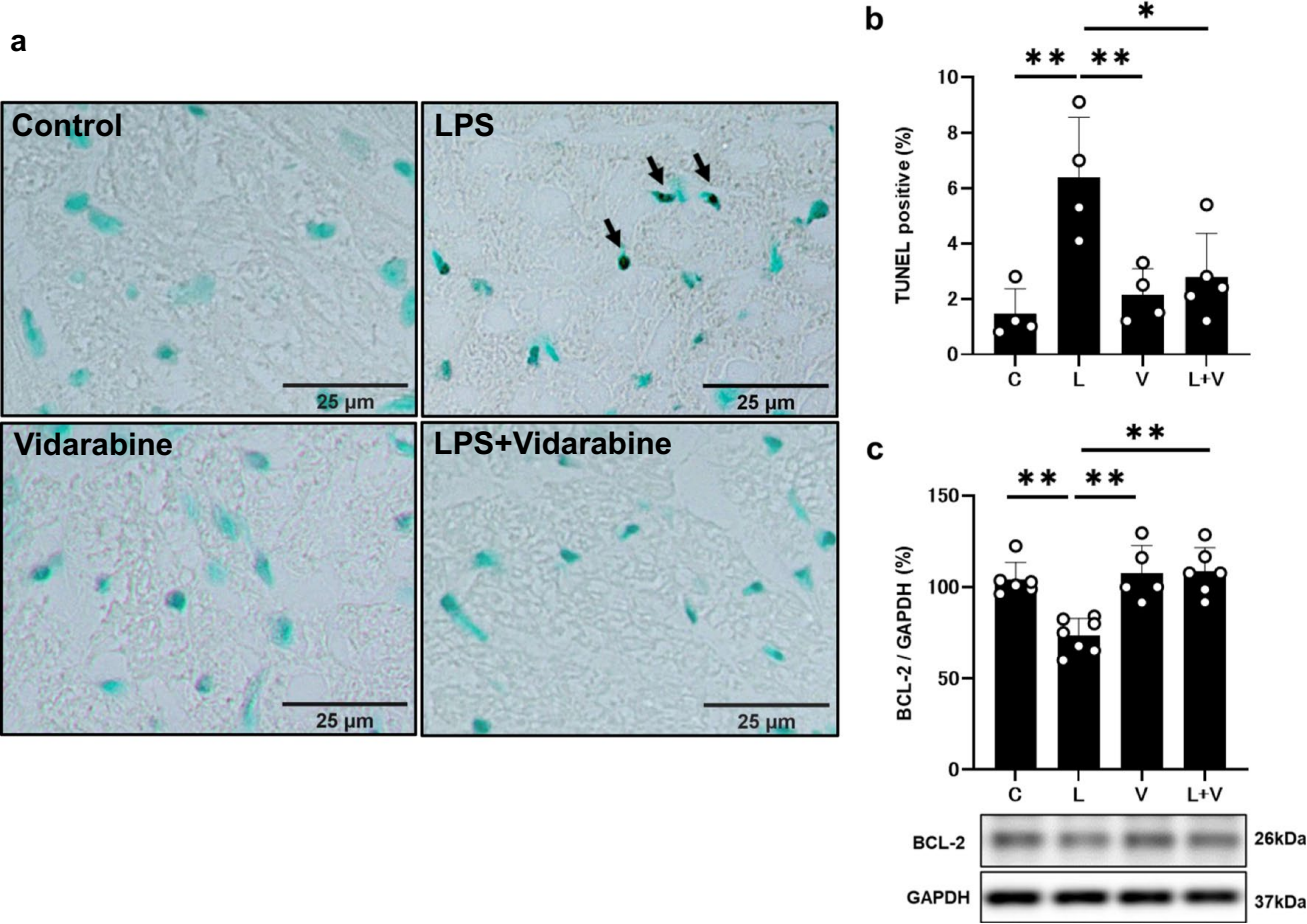
Effects of vidarabine on PG-LPS-induced cardiac myocyte apoptosis. **a** Representative images of TUNEL-stained sections of cardiac muscle in the Control (*upper left*), PG-LPS (*upper right*), vidarabine (*lower left*), and PG-LPS + vidarabine (*lower right*) groups. Scale bar: 25 μm. **b** The number of TUNEL-positive myocyte was significantly increased in the PG-LPS group (*n* = 4, **P < 0.01), but this increase was blocked in the PG-LPS + vidarabine group (*n* = 5, **P < 0.01). One-way ANOVA followed by Tukey’s post hoc test). **c** Expression of anti-apoptotic BCL-2 protein was significantly decreased in the cardiac muscle of PG-LPS group (*n* = 7, **P < 0.01), but this change was significantly blocked in the cardiac muscle of PG-LPS + vidarabine group (*n* = 5, *P < 0.05). One-way ANOVA followed by Tukey’s post hoc test). Full-size images of immunoblots are presented in Additional file 1: Fig. S2. Data are presented as mean ± SD and scattered dots show individual data

We next examined apoptosis of cardiac myocytes in PG-LPS-treated mice with/without vidarabine by means of TUNEL staining (Fig. 3a). PG-LPS treatment significantly increased apoptosis (Control [*n* = 4] vs. PG-LPS [*n* = 4]: 1.5 ± 0.9 vs. 6.4 ± 2.2%, *P* < 0.01 by one-way ANOVA followed by Tukey’s post hoc test). Vidarabine alone (*n* = 4) had no effect on the number of TUNEL-positive cardiac myocytes, but it blocked the PG-LPS-induced increase of TUNEL-positive cardiac myocytes (PG-LPS [*n* = 4] vs. PG-LPS + vidarabine [*n* = 5]: 6.4 ± 2.2 vs. 2.8 ± 1.6%, *P* < 0.05 by one-way ANOVA followed by the Tukey’s post hoc test) (Fig. 3b).

We also evaluated apoptosis of cardiac myocytes by measuring the change of BCL-2, a regulator of apoptosis, in the heart (Fig. 3c and Additional file 1: Fig. S2). BCL-2 expression was significantly decreased in cardiac muscle of PG-LPS-treated mice (Control [*n* = 6] vs. PG-LPS [*n* = 7]: 100 ± 9.3 vs. 74 ± 9.3%, *P* < 0.01 by one-way ANOVA followed by Tukey’s post hoc test) and the increase was significantly attenuated by vidarabine (PG-LPS [*n* = 7] vs. PG-LPS + vidarabine [*n* = 6]: 74 ± 9.3 vs. 109 ± 13%, *P* < 0.01 by one-way ANOVA followed by Tukey` post hoc test).

### Effects of PG-LPS on AC5 expression with/without vidarabine

**Fig. 4:**
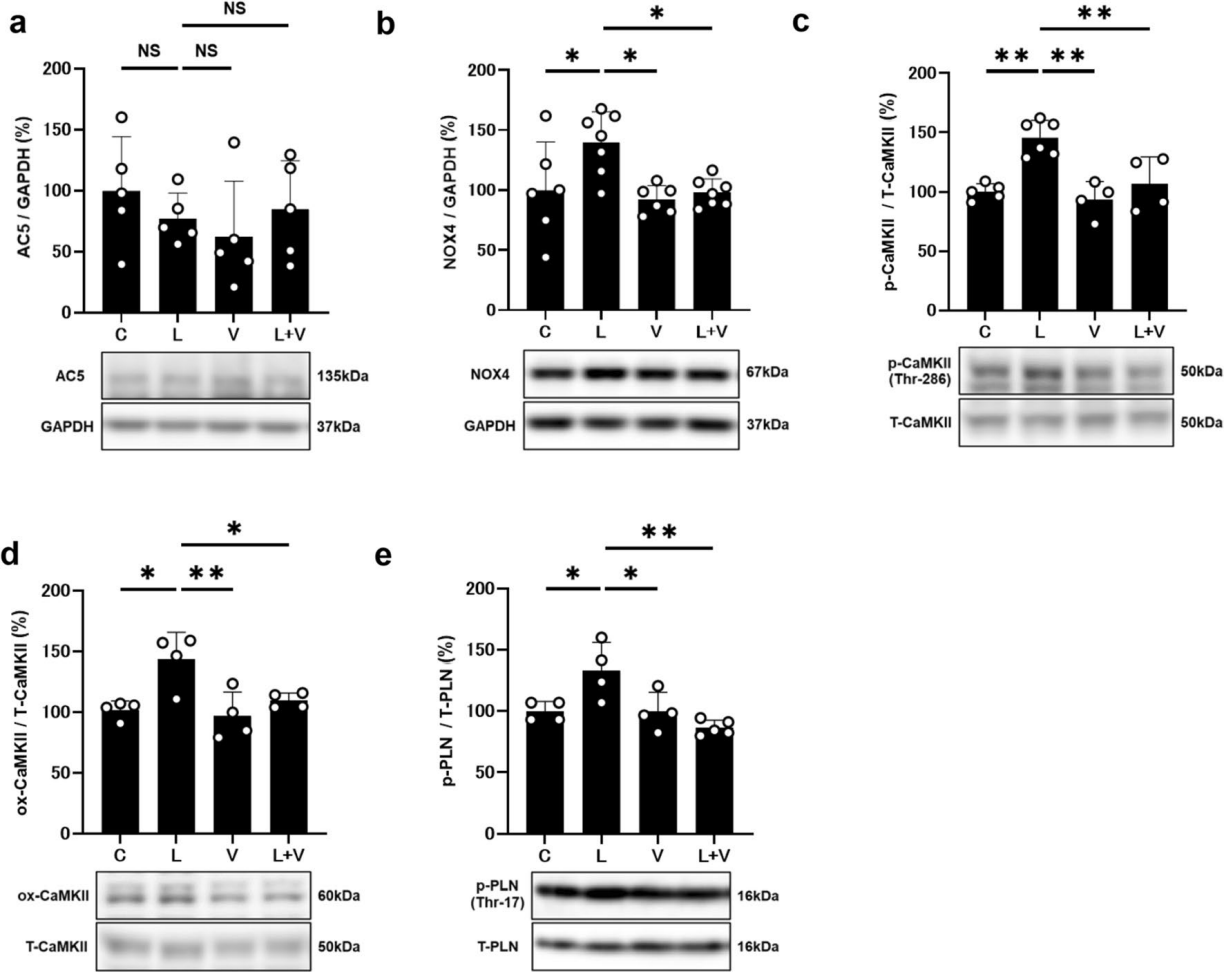
Effects of vidarabine on AC5, NOX4, p-CaMKII, ox-CaMKII and phospho-PLN. **a** AC5 expression was similar in cardiac muscle of all four groups. NS; not significantly different. One-way ANOVA followed. by Tukey’s post hoc test) Full-size images of immunoblots are presented in Additional file 1: Fig. S3. **b** NOX4 expression was significantly increased in the cardiac muscle of PG-LPS group (*n* = 7, *P < 0.05), and this increase was significantly blocked in the PG-LPS + vidarabine group (*n* = 7, *P < 0.05). One-way ANOVA followed by Tukey’s post hoc test). Full-size images of immunoblots are presented in Additional file 1: Fig. S4. **c** Expression of phospho-CaMKII (Thr-286) was significantly increased in the PG-LPS group (*n* = 6, **P < 0.01), and this increase was significantly attenuated in the PG-LPS + vidarabine group (n = 7, **P < 0.01). One-way ANOVA followed by Tukey’s post hoc test). Full-size images of immunoblots are presented in Additional file 1: Fig. S5. **d** Expression of oxidized-CaMKII (ox-CaMKII) was significantly increased in the PG-LPS group (*n* = 4, *P < 0.01) and this increase was significantly attenuated in the PG-LPS + vidarabine group (*n* = 4, *P < 0.05). One-way ANOVA followed by the Tukey–Kramer post hoc test. Full-size images of immunoblots are presented in Additional file 1: Fig. S6. **e** Expression of phospho-PLN (Thr-17) was significantly increased in the PG-LPS group (*n* = 4, *P < 0.05), and this increase was significantly attenuated in the PG-LPS + vidarabine group (*n* = 5, *P < 0.01). One-way ANOVA followed by Tukey’s post hoc test). Full-size images of immunoblots are presented in Additional file 1: Fig. S7. Data are presented as mean ± SD and scattered dots show individual data

Increased AC5 expression was previously demonstrated in heart failure induced by chronic catecholamine stress [13]. We thus examined the expression of AC5 in the heart and found similar levels among the four groups (Fig. 4a and Additional file 1: Fig. S3).

### Effects of PG-LPS on NOX4 expression with/without vidarabine

NOX4 expression was significantly increased in the PG-LPS-treated group (Control [*n* = 6] vs. PG-LPS [*n* = 7]: 100 ± 40.2 vs. 140 ± 25.9%, *P* < 0.05 vs. Control), and the increase was suppressed by vidarabine (PG-LPS [*n* = 7] vs. PG-LPS + vidarabine [*n* = 7]; 140 ± 25.9 vs. 98 ± 11.6%, *P* < 0.05 vs. PG-LPS) (Fig. 4b and Additional file 1: Fig. S4).

### Effects of PG-LPS on CaMKII phosphorylation with/without vidarabine

CaMKII is activated via phosphorylation and oxidization in the presence of ROS and contributes to the development of cardiac remodeling and dysfunction [35]. We thus examined the amounts of phospho-CaMKII (Thr-286) (Fig. 4c and Additional file 1: Fig. S5) and oxidized methionine-281/282 CaMKII (ox-CaMKII) in the heart in the four groups (Fig. 4d and Additional file 1: Fig. S6) and found that they were significantly increased at 1 week after PG-LPS treatment (phospho-CaMKII (Thr-286): Control [*n* = 5] vs. PG-LPS [*n* = 6]: 100 ± 6.9 vs. 146 ± 14.6%, *P* < 0.01 vs. Control; ox-CaMKII: Control [*n* = 4] vs. PG-LPS [*n* = 4]: 100 ± 7.5 vs. 143 ± 22.4%, *P* < 0.05 vs. Control). These changes were significantly attenuated by vidarabine (phospho-CaMKII (Thr-286): PG-LPS [*n* = 6] vs. PG-LPS + vidarabine [*n* = 4]; 146 ± 14.6 vs. 107 ± 22.6%, *P* < 0.01 vs. PG-LPS; ox-CaMKII: PG-LPS [*n* = 4] vs. PG-LPS + vidarabine [*n* = 4]; 143 ± 22.4 vs. 110 ± 6.3%, *P* < 0.05 vs. PG-LPS).

### Effects of PG-LPS on PLN phosphorylation with/without vidarabine

Phospho-PLN (Thr-17) was significantly increased in the heart of the PG-LPS-treated group (Control [*n* = 4] vs. PG-LPS [*n* = 4]: 100 ± 7.9 vs. 133 ± 22%, *P* < 0.05 vs. Control). This increase was significantly attenuated by vidarabine (PG-LPS [*n* = 4] vs. PG-LPS + vidarabine [*n* = 5]; 133 ± 22 vs. 86 ± 6.1%, *P* < 0.01 vs. PG-LPS) (Fig. 4e and Additional file 1: Fig. S7).

## Discussion

Our findings here indicate that cardiac function was significantly impaired in mice treated with PG-LPS at a dose consistent with circulating levels in PD patients, and myocyte apoptosis and fibrosis were significantly increased. Importantly, these changes were blunted by pharmacological inhibition of AC5. We also investigated the mechanism of these changes.

It is well established that sustained sympathetic overactivity is associated with the development of end-organ damage, including cardiac remodeling and cardiac dysfunction [36]. Importantly, we and another group previously demonstrated that genetic disruption and pharmacological inhibition of AC5 might be associated with resistance to the cardiac remodeling and cardiac dysfunction after chronic catecholamine stress, probably via activation of the mitogen/extracellular signal-regulated kinase/extracellular signal-regulated kinase signaling pathway and upregulation of cell-protective molecules, including superoxide dismutase [13, 37, 38]. In addition, we have previously demonstrated that inhibition of AC5 with vidarabine attenuates adrenergic receptor stimulation-induced Ca^2+^ leakage and spontaneous Ca^2+^ release from SR, as well as sympathetic activation-induced ROS production in isolated cardiac myocyte [14], which is involved in various physiological and pathological processes in the heart, including fibrosis, apoptosis and heart failure [15, 38].

**Fig. 5:**
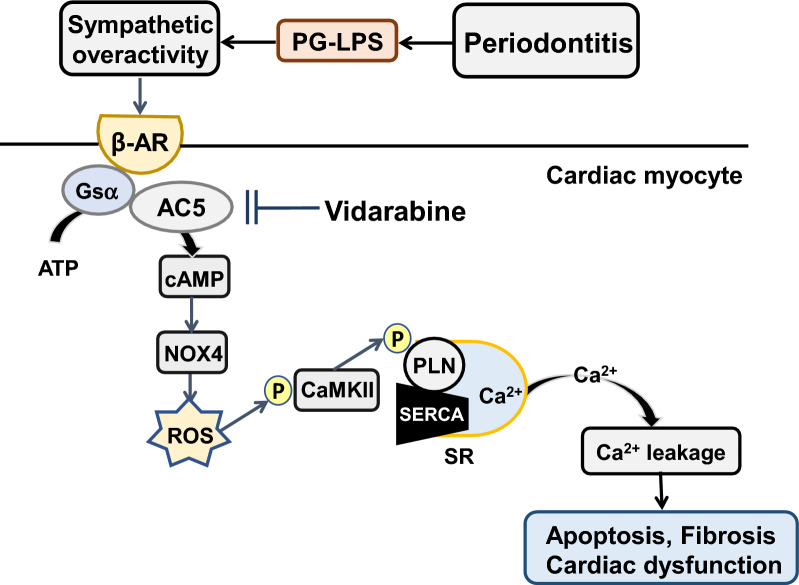
This scheme illustrates the proposed role of AC5 in the heart of PG-LPS-treated mice. Mice with experimental periodontitis induced by the ligation of the left first molar [39] or by chronic PG-LPS infusion as used in this study [18] show sympathetic overactivity. β-AR/G_S_α/AC5 signaling is activated by PG-LPS treatment, leading to ROS production via NOX4 and PLN phosphorylation at Thr-17. These changes might cause fibrosis and myocyte apoptosis in the heart of PG-LPS-treated mice, leading to cardiac dysfunction

Mice with PD induced by the ligation of the left first molar [39] or chronic PG-LPS infusion as used in this study [18] show sympathetic overactivity (Fig. 5). Although acute sympathetic stimulation and activation of the cAMP-PKA pathway play a major role in improving cardiac function, previous studies using transgenic models have demonstrated that chronic sympathetic overactivity caused by the cardio-specific overexpression of β-AR [40, 41], Gsα [42], PKA [43] or CaMKII [44] resulted in cardiomyopathy. We previously showed that mice with disruption of AC5 exhibited attenuated responses to chronic sympathetic activation, indicating that AC5 might play an important role in the development of cardiac disruption in response to chronic sympathetic stimulation [11–13].

We recently established that activation of toll-like receptor 4 signaling in mice induced by PG-LPS at the dose used in this study causes cardiac dysfunction, myocyte apoptosis and fibrosis in cardiac muscle, leading to abundant production of ROS and Ca^2+^ leakage from sarcoendoplasmic reticulum due to CaMKII-mediated phosphorylation of PLN (at Thr-17) [20]. We also demonstrated that the cAMP-CaMKII pathway is activated in mice treated with PG-LPS, as used in this study [18]. Our present data, together with the previous findings, suggest that AC5 plays an important role in the development of PG-LPS-mediated cardiac dysfunction, and thus AC5 might be a therapeutic target for the treatment of cardiac dysfunction in patients with PD.

We recently reported that occlusal-disharmony-induced CVD might arise, at least in part, through the upregulation of NOX4 induced by activation of AC5 [21]. In addition, we recently demonstrated that NOX4 expression was significantly increased in the heart of PG-LPS-treated mice at the dose used in this study for 4 weeks, and this increase was effectively alleviated by pharmacological inhibition of Toll-like receptor 4 (TLR4), a target for PG-LPS, with TLR4 antagonist (6R)-6-[*N*-(2-chloro-4-fluorophenyl)sulfamoyl] cyclohex-1-ene-1-carboxylate (TAK-242) [20], suggesting that activation of TLR4 via PG-LPS might play a role, at least in part, in the increased NOX4 expression in the heart of PG-LPS-treated mice. In this study, we demonstrated that expression of NOX4 was also increased in the heart of mice treated with PG-LPS at a dose equivalent to the circulating levels in PD patients. Importantly, ROS production in the oral cavity might cause not only local pathogenic disturbance, but also systemic diseases, including CVD [45, 46]. Our current findings, together with the previous studies, indicate that ROS generation via NOX4 might affect not only cardiac function, but also general health and mortality (Fig. 5).

Chronic sympathetic overactivity induces activation of cardiac AC subtypes, thereby increasing intracellular cAMP concentration. Furthermore, activation of PKA and CaMKII has been reported to increase cardiac myocyte apoptosis and cardiac dysfunction due to PLN phosphorylation, which leads to Ca^2+^ leakage (Fig. 5) [47, 48]. We previously demonstrated that occlusal disharmony might cause cardiac dysfunction through the activation of sympathetic nerve activity, and a β-AR blocker prevented occlusal-disharmony-induced cardiac dysfunction [22, 49]. Our current results suggest that β-AR blockers might be useful for the treatment of CVD in patients with periodontal disease, as in the case of occlusal disharmony.

However, β-AR blockers have several critical side effects. The inhibition of sympathetic signaling reduces cardiac function. In addition, great caution is required in the use of β-AR blockers for the treatment of CVD in aged patients, because β-AR is expressed in the pulmonary bronchus and pancreas, so that inhibition may lead to bronchospasms and glucose intolerance [26, 50]. In this study, we demonstrated that vidarabine inhibits the development of PG-LPS-mediated cardiac dysfunction without suppressing cardiac function at a dose used clinically in humans, in contrast to β-blocker administration [15, 21]. This study indicates that the use of vidarabine to suppress only the activity of AC5, but not the entire β-AR signaling pathway, may be preferable to β-AR blockade therapy for the treatment of CVD associated with periodontal disease (Fig. 5).

## Conclusion

Our current and previous studies suggest that vidarabine might broadly inhibit oral frailty-mediated cardiomyopathy, leading to improved longevity and reduced physical frailty. Importantly, an early clinically trial should be feasible because vidarabine is a clinically approved drug.

### Supplementary Information

**Additional file 1: Fig. S1. **Representative full-length immunoblots of Fig. 2c. The amount of α-SMA (*left panel*) and GAPDH (*right panel*) were shown. The black-line box indicated by arrow in each blot is corresponded to the cropped parts that are showed in the main article. **Fig. S2. **Representative full-length immunoblots of Fig. 3c. The amount of BCL-2 (*left panel*) and GAPDH (*right panel*) were shown. The black-line box indicated by arrow in each blot is corresponded to the cropped parts that are showed in the main article. **Fig. S3. **Representative full-length immunoblots of Fig. 4a. The amount of AC5 (*left panel*) and GAPDH (*right panel*) were shown. The black-line box indicated by arrow in each blot is corresponded to the cropped parts that are showed in the main article. **Fig. S4. **Representative full-length immunoblots of Fig. 4b. The amount of NOX4 (*left panel*) and GAPDH (*right panel*) were shown. The black-line box indicated by arrow in each blot is corresponded to the cropped parts that are showed in the main article. **Fig. S5. **Representative full-length immunoblots of Fig. 4c. The amount of p-CaMKII (Thr-286) (*left panel*) and total-CaMKII (*right panel*) were shown. The black-line box indicated by arrow in each blot is corresponded to the cropped parts that are showed in the main article. **Fig. S6. **Representative full-length immunoblots of Fig. 4d. The amount of ox-CaMKII (*left panel*) and total (*right panel*) were shown. The black-line box indicated by arrow in each blot is corresponded to the cropped parts that are showed in the main article. **Fig. S7. **Representative full-length immunoblots of Fig. 4e. The amount of p-PLN (Thr-17) (*left panel*) and total-PLN (*right panel*) were shown. The black-line box indicated by arrow in each blot is corresponded to the cropped parts that are showed in the main article.

## Data Availability

The datasets used and/or analyzed during the current study are available from the corresponding author on reasonable request.

## Abbreviations

AC5: Type 5 adenylyl cyclase
PG-LPS: *Porphylomonas gingivalis* lipopolysaccharide
PD: Periodontitis
CVD: Cardiovascular disease
β-AR: β-Adrenergic receptor
AC: Adenylyl cyclase
AC5: Type 5 adenylyl cyclase
ROS: Reactive oxygen species
NOX4: Nicotinamide adenine dinucleotide phosphate oxidase 4
cAMP: Cyclic AMP
PKA: Protein kinase A
CaMKII: Ca^2+^/calmodulin-dependent protein kinase II
PLN: Phospholamban
PBS: Phosphate buffered saline
i.p.: Intraperitoneal
Control: Control group
BW: Body weight
TL: Tibial length
LV: Left ventricle
HR: Heart rate
EDV: Left ventricular end-diastolic volume
ESV: Left ventricular end-systolic volume
LVIDd: Left ventricular internal dimension at end-diastole
LVIDs: Left ventricular internal dimension at end-systole
SV: Stroke volume
CO: Cardiac output
EF: Left ventricular ejection fraction
%FS: Left ventricular fractional shortening
TUNEL: Terminal deoxyribonucleotidyl transferase (TdT)-mediated biotin-16-deoxyuridine triphosphate (dUTP) nick-end labeling
α-SMA: α-Smooth muscle actin
BCL-2: B cell lymphoma 2
GAPDH: Glyceraldehyde 3-phosphate dehydrogenase
SD: Standard deviation
TLR4: Toll-like receptor 4
