# Supplementary material for: Vidarabine, an anti-herpes agent, improves Porphyromonas gingivalis lipopolysaccharide-induced cardiac dysfunction in mice
Source: J Physiol Sci. 2023 Aug 9;73:18. doi: 10.1186/s12576-023-00873-5 (PMC10717078; doi:10.1186/s12576-023-00873-5)
Supplement: Supplementary file 1 — Additional file 1: Fig. S1. Representative full-length immunoblots of Fig. 2c. The amount of α-SMA (left panel) and GAPDH (right panel) were shown. The black-line box indicated by arrow in each blot is corresponded to the cropped parts that are showed in the main article. Fig. S2. Representative full-length immunoblots of Fig. 3c. The amount of BCL-2 (left panel) and GAPDH (right panel) were shown. The black-line box indicated by arrow in each blot is corresponded to the cropped parts that are showed in the main article. Fig. S3. Representative full-length immunoblots of Fig. 4a. The amount of AC5 (left panel) and GAPDH (right panel) were shown. The black-line box indicated by arrow in each blot is corresponded to the cropped parts that are showed in the main article. Fig. S4. Representative full-length immunoblots of Fig. 4b. The amount of NOX4 (left panel) and GAPDH (right panel) were shown. The black-line box indicated by arrow in each blot is corresponded to the cropped parts that are showed in the main article. Fig. S5. Representative full-length immunoblots of Fig. 4c. The amount of p-CaMKII (Thr-286) (left panel) and total-CaMKII (right panel) were shown. The black-line box indicated by arrow in each blot is corresponded to the cropped parts that are showed in the main article. Fig. S6. Representative full-length immunoblots of Fig. 4d. The amount of ox-CaMKII (left panel) and total (right panel) were shown. The black-line box indicated by arrow in each blot is corresponded to the cropped parts that are showed in the main article. Fig. S7. Representative full-length immunoblots of Fig. 4e. The amount of p-PLN (Thr-17) (left panel) and total-PLN (right panel) were shown. The black-line box indicated by arrow in each blot is corresponded to the cropped parts that are showed in the main article. [file 12576_2023_873_MOESM1_ESM.pdf]

Additional File 1

Vidarabine, an anti-herpes agent, improves *Porphyromonas gingivalis* lipopolysaccharide-induced cardiac dysfunction in mice

Running title: Role of AC5 in the heart of periodontis

Michinori Tsunoda [1,2¶], Ichiro Matsuo [2¶], Yoshiki Ohnuki [1], Kenji Suita [1], Misao Ishikawa [3], Aiko Ito [4], Yasumasa Mototani [1], Kenichi Kiyomoto [1,2], Akinaka Morii [1,2], Megumi Nariyama [5], Yoshio Hayakawa [6], Kazuhiro Gomi [2], Satoshi Okumura [1]

[1] Department of Physiology, Tsurumi University School of Dental Medicine, Yokohama 230-8501, Japan

[2] Department of Periodontology, Tsurumi University School of Dental Medicine, Yokohama 230-8501, Japan

[3] Department of Oral Anatomy, Tsurumi University School of Dental Medicine, Yokohama 230-8501, Japan

[4] Department of Orthodontology, Tsurumi University School of Dental Medicine, Yokohama 230-8501, Japan

[5] Department of Pediatric Dentistry, Tsurumi University School of Dental Medicine, Yokohama 236-8501, Japan

[6] Department of Dental anesthesiology, Tsurumi University School of Dental Medicine,

Yokohama 230-8501, Japan

¶ These authors contributed equally to this work.

*****Corresponding author: Satoshi Okumura:

Department of Physiology, Tsurumi University School of Dental Medicine,

2-1-3 Tsurumi, Tsurumi-ku, Yokohama 230-8501; (Tel. +81-(0)45-580-8476;

Fax. +81-(0)45-585-2889; e-mail: okumura-s@tsurumi-u.ac.jp)

Figure S1

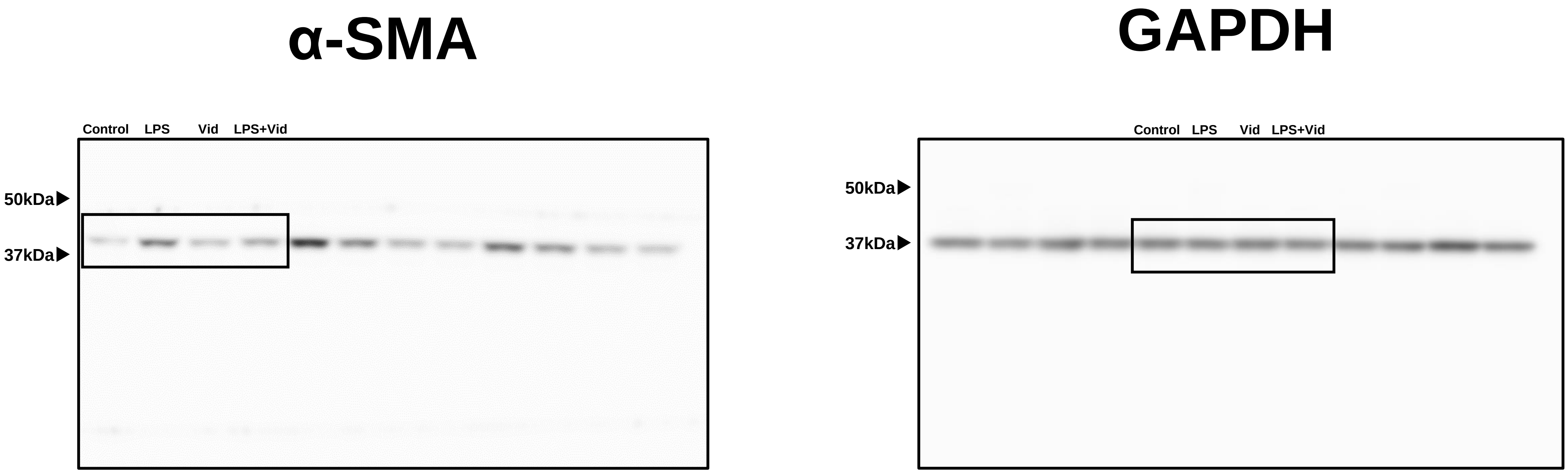

Fig. S1

Representative full-length immunoblots of **Fig. 2c**. The amount of α-SMA **(*left panel*)** and GAPDH **(*right panel*)** were shown. The black-line box indicated by arrow in each blot is corresponded to the cropped parts that are showed in the main article.

Figure S2

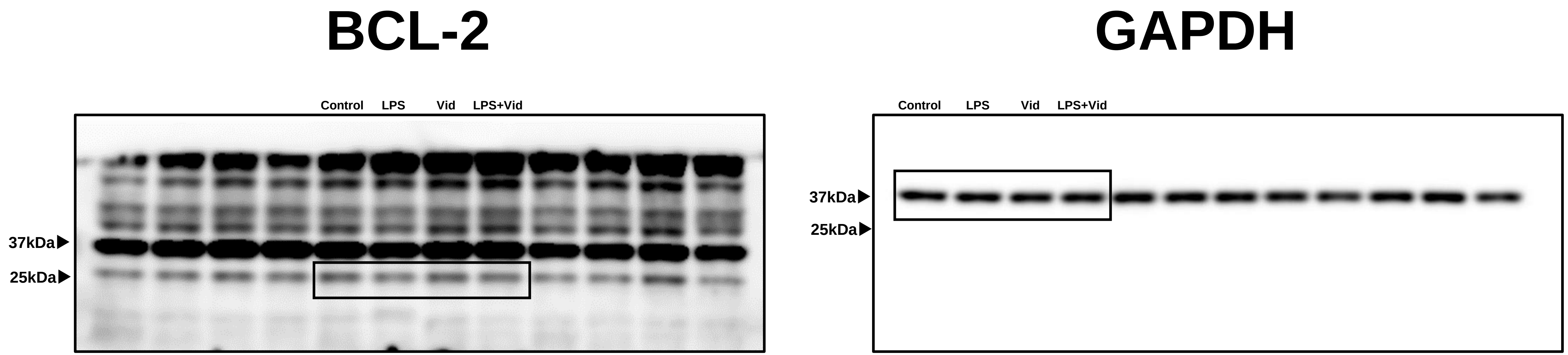

Fig. S2
Representative full-length immunoblots of **Fig. 3c**. The amount of BCL-2 **(*left panel*)** and GAPDH **(*right panel*)** were shown. The black-line box indicated by arrow in each blot is corresponded to the cropped parts that are showed in the main article.

Figure S3

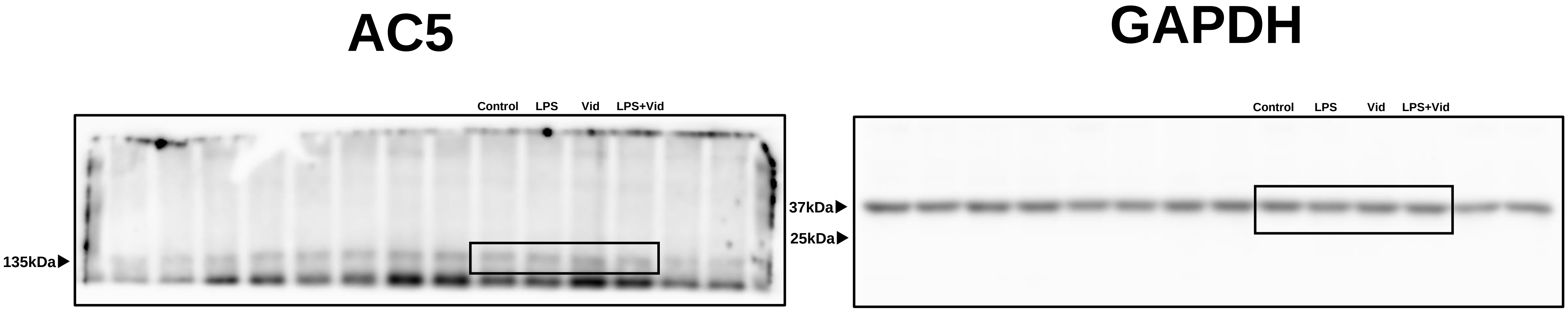

Fig. S3
Representative full-length immunoblots of **Fig. 4a**. The amount of AC5 **(*left panel*)** and GAPDH **(*right panel*)** were shown. The black-line box indicated by arrow in each blot is corresponded to the cropped parts that are showed in the main article.

Figure S4

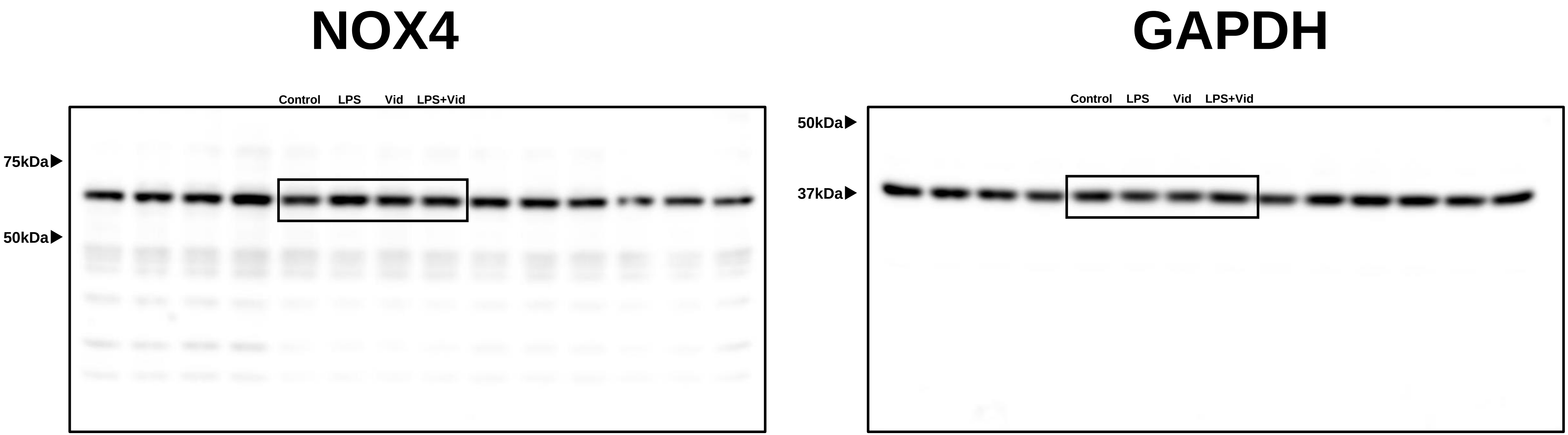

Fig. S4

Representative full-length immunoblots of **Fig. 4b**. The amount of NOX4 **(*left panel*)** and GAPDH **(*right panel*)** were shown. The black-line box indicated by arrow in each blot is corresponded to the cropped parts that are showed in the main article.

Figure S5

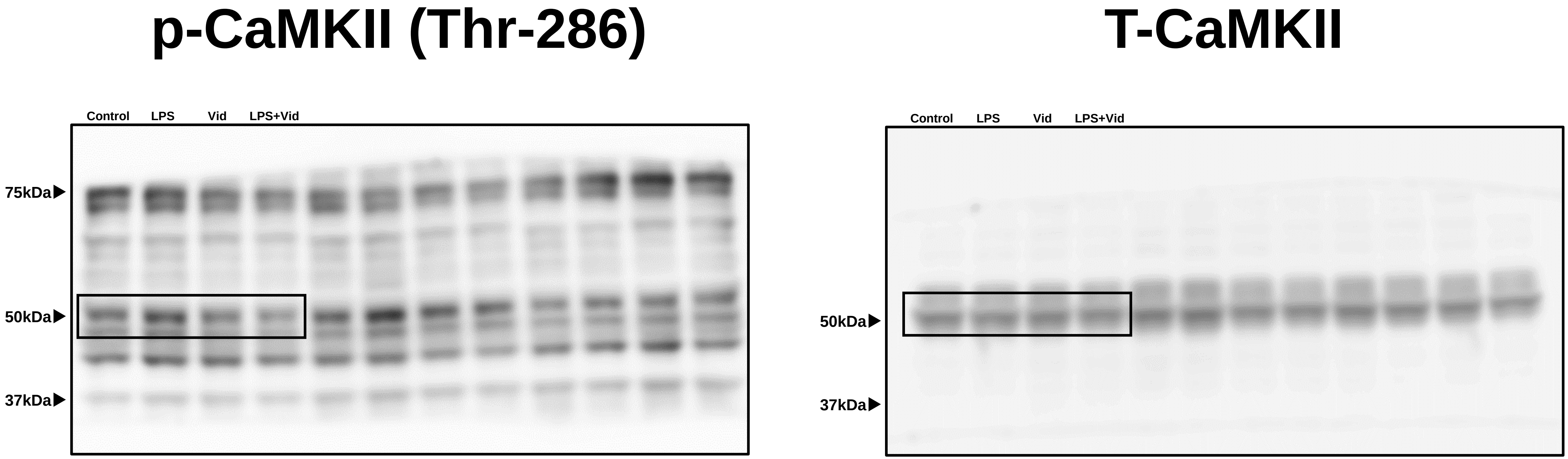

Fig. S5

Representative full-length immunoblots of **Fig. 4c**. The amount of p-CaMKII (Thr-286) **(*left panel*)** and total-CaMKII **(*right panel*)** were shown. The black-line box indicated by arrow in each blot is corresponded to the cropped parts that are showed in the main article.

Figure S6

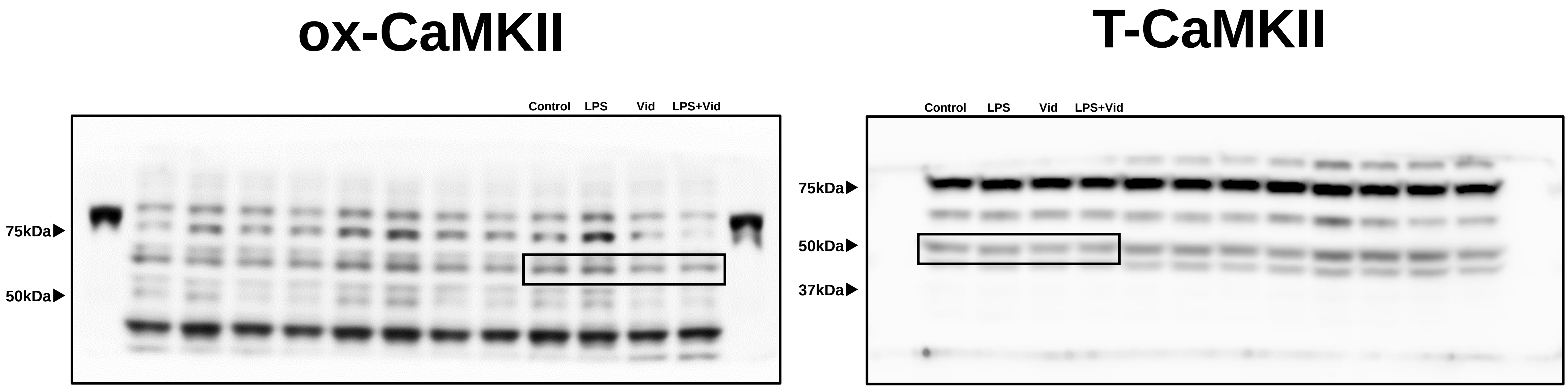

Fig. S6

Representative full-length immunoblots of **Fig. 4d**. The amount of ox-CaMKII **(*left panel*)** and total **(*right panel*)** were shown. The black-line box indicated by arrow in each blot is corresponded to the cropped parts that are showed in the main article.

Figure S7

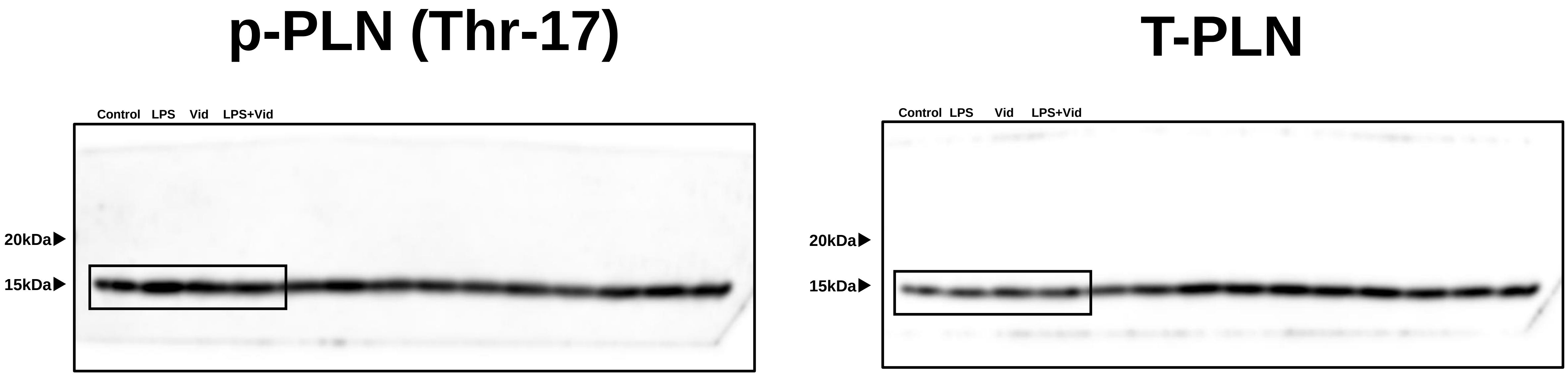

Fig. S7
Representative full-length immunoblots of **Fig. 4e**. The amount of p-PLN (Thr-17) **(*left panel*)** and total-PLN **(*right panel*)** were shown. The black-line box indicated by arrow in each blot is corresponded to the cropped parts that are showed in the main article.
